# Pathogenomics of Uterine Fibroids Development

**DOI:** 10.3390/ijms20246151

**Published:** 2019-12-06

**Authors:** Vladislav S. Baranov, Natalia S. Osinovskaya, Maria I. Yarmolinskaya

**Affiliations:** D.O. Ott Research Institute of Obstetrics, Gynecology and Reproductology, 199034 Saint-Petersburg, Russia; natosinovskaya@mail.ru (N.S.O.); m.yarmolinskaya@gmail.com (M.I.Y.)

**Keywords:** uterine leiomyoma, stem cells, molecular mechanisms, pathogenomics, gene networks, driver mutations

## Abstract

We review recent studies dealing with the molecular genetics and basic results of omics analysis of uterine leiomyoma (LM)—a common benign muscle tumor of the uterus. Whole genome studies of LM resulted in the discovery of many new gene nets and biological pathways, including its origin, transcriptomic, and epigenetic profiles, as well as the impact of the inter-cell matrix in LM growth and involvement of microRNA in its regulation. New data on somatic cell mutations ultimately involved in the origin, distribution and growth of LM are reviewed. Putative identification of LM progenitor SC (stem cells) giving rise to maternal fibroid nodes and junctional zones provide a new clue for hypotheses on the pathogenomics of LM. The reviewed data are consistent with at least two different but probably intimately interacted molecular mechanisms of LM. One of them (the genetic hypothesis) is focused primarily on the *MED12* gene mutations and suggests its onset in the side population of embryonic myoblasts of the female reproductive system, which later gave rise to multiple small and medium fibroids. The single and usually large-size fibroids are induced by predominantly epigenetic disorders in LM SC, provoked by enhanced expression of the *HMGA2* gene caused by its hypomethylation and epigenetic deregulation enhanced by hypoxia, muscle tension, or chromosome instability/aberrations. The pathogenomics of both genetic and epigenetic programs of LM with many peculiarities at the beginning later became rather similar and partly overlapped due to the proximity of their gene nets and epigenetic landscape. Pathogenomic studies of LM open ways for elaboration of novel strategies of prevention and treatment of this common disease.

## 1. Introduction

Leiomyoma (LM) (fibromyoma)—a common benign tumor of the uterus—is an important medical and social problem, given the high incidence (up to 40% in women of reproductive age) [1,2,3]. The causes and pathogenetic mechanisms of the development of leiomyomas have not been fully established. The widespread introduction of molecular genetic research methods into modern medicine has made it possible to obtain fundamentally new information on the pathomorphological and hormonal causes of the onset and development of LM, as well as on the characteristics of the genome, indicating an important role of heredity in the development of the disease. Genetic factors and molecular mechanisms of pathogenesis of LM have been repeatedly discussed in the scientific literature [3,4,5]. Recent studies searching for the parental (progenitor) cells of LM, as well as systemic (omix) analysis of LM, have significantly deepened and expanded the understanding of the pathogenomics of LM, creating real prerequisites for the development of new promising models of the disease and its targeted therapy. New data on the pathogenesis of LM obtained in recent years by genomic medicine, including using omix technology, is reviewed here.

## 2. The Origin of Fibroids

Fibroid progenitor cells remained unknown for a long time [6,7,8]. In 2013, cells with an unusual asymmetric division were identified in experiments using 5-bromo-2-deoxyuridine (BrdU) cell markers in myoblast culture. As a result of this “asymmetric” division, two populations of cells arise: Progenitor myoblasts and lateral populations (side populations)—undifferentiated additional cells. The former differentiates into smooth muscle cells—myocytes—and the latter retains the ability for asymmetric division, which is a characteristic of stem cells (SC) [9,10]. It has been established that cells of the additional population under hypoxic conditions actively divide and express the stem cell genes typical of embryonic/progenitor myometrial cells: *OCT-4, NANOG, DNMT3B*, and *GDF3* [11]. The origin of mesenchymal cells is evidenced by their ability to differentiate in vitro into adipocytes and osteocytes. The fraction of such SCs in myomas is not large and amounts to about 1% [10]. According to updated data, myometrium stem cells that carry the antigens CD45(−), CD31(−), glycophorin A(−), CD49f (+), and CD34(+) are involved in the onset and development of LM, and the genes *OCT-4* and *NANOG* are expressed and are capable of differentiating into myoblasts, osteoblasts, lipocytes, chondrocytes, and other mesenchymal derivatives [12,13]. The presence of SC in myometrium has recently been directly confirmed in transgenic mice with the *OCT-4* gene using a new method for detecting the surface marker Stro1/CD44 [14].

## 3. Tumor Initiation

The precipitating factors and molecular mechanisms of tumor transformation of the smooth muscle cells of LM are actively being studied. It is known that an important role in this is played by various stressful effects, especially hypoxia and muscle contractions of the uterus during menstruation, pregnancy, and childbirth [15]. Groups of SC LM can appear in various layers of the myometrium: In the subserous, in the submucous, and in the intramural [11]. Most often, they occur at the interface of the endometrium and the myometrium in the so-called border zone (junctional zone) [16]. LM tissue is weakly vascularized, contains bundles of smooth muscle fibers and extracellular matrix material (ECM) formed by collagen, fibronectin, laminin, and proteoglycans. Mechanotransduction is a process that allows cells to adapt to a changing physical environment, perceiving the environment and translating mechanical stress into biochemical signals [17]. Structural and functional disorders of ECM contribute to the growth of LM, which opens up opportunities for finding new ways to treat this disease [18]. As the tumor grows, the volume of ECM progressively increases, which leads to the transmission of signals from ECM to the cell through integrins and mediators, the inclusion of p38MAPK/ERK signaling cascades, and conserved signaling pathways, which coordinately regulate diverse cellular activities and is accompanied by impaired expression of the *TGF-β* (transforming growth factor β), *ACVR1* (activin A receptor, type I), *PDGF* (platelet-derived growth factors), and *TNF-α* (tumor necrosis factor-alpha) genes that regulate the metabolism of steroid hormones estrogen and progesterone. In this case, the expression profile of regulatory microRNAs (miR-29, miR-200c, and miR-93/106b) changes significantly. Disorder expression relates primarily to regulatory genes (*TGF-β* and *ACVR1*), which determine the characteristics of the interaction of fibroid cells and the intercellular matrix. Given the crucial role of ECM in the development of LM, effective control of its size with the help of drugs is considered as a new promising strategy for drug therapy of LM [16].

The growth of myometrium and connective tissue suggests the presence of appropriate SCs that have recently been identified [12,13]. It is SCs that determine the growth in the number of myometrial cells, an increase in the volume of ECM, and participate in the processes of uterine remodeling during pregnancy. It is important to note that mature smooth muscle fiber cells (myocytes) contain a large number of estrogen receptors ά (ERα) and progesterone receptors (PR). These receptors, however, are absent on the membranes of SCs themselves. It has been established that paracrine factors, including steroid hormones and Wnt ligands secreted by myocytes and fibrous cells, reach SC, stimulate their division, and direct their differentiation into LM cells [1].

The decisive role in the initiation of tumor growth and the appearance of different types of LM are determined by genetic factors [10]. There is no doubt that LM is a heterogeneous group of benign tumors, both in relation to the molecular mechanisms of pathogenesis and the ways of implementing them. The main drivers of LM are structural and functional changes in the SC genome. So, it is well known that 25% to 40% of all LMs have various abnormalities of the karyotype, including translocation and deletions, as well as more complex disorders, such as chromothripsis (rearrangement of chromosome fragments within one or between several different chromosomes) [19,20,21,22]. Cytogenetic rearrangements characteristic of LM include translocation between chromosomes 12 and 14, trisomy 12, deletions of the sections of the long arm of chromosomes 3 or 7 and the short arm of chromosome 1, as well as rearrangements of the short arm of chromosome 6 and chromosomes 1, 3, 10, 13 and X [23]. Various submicroscopic changes in the genome are often found in LM cells, which are detected, inter alia, by the method of comparative genomic hybridization (CGH) [24].

The main genetic drivers of LM include the *MED12*, *HMGA2*, *FH*, and *COL4A5*-*COL4A6* genes [25].

1. The *MED12* gene encodes one of the proteins of the mediator complex involved in the regulation of the activity of the key transcription enzyme, RNA polymerase 2. Somatic mutations of the *MED12* gene are detected in 70%–75% of patients with LM [26,27] and arise de novo directly in myoblasts. In addition to LM cells, *MED12* gene mutations also occur in adenomyosis (internal endometriosis) [28] and in mammary adenomyoma cells [25]. When mutations of the *MED12* gene occur in ontogeny remains unknown.

2. The *HMGA2* gene encodes a non-histone chromatin protein that belongs to high mobility group proteins, and regulates transcription processes. The *HMGA2* gene contains three DNA-binding domains (AT hooks), through which a protein binds to nuclear DNA at loci rich in AT dinucleotides. *HMGA2* is involved in the assembly of protein complexes that regulate gene transcription. With rare exceptions, *HMGA2* gene expression is high during embryogenesis and drops sharply after birth [29]. The expression level of the *HMGA2* gene depends on the dinucleotide repeat (TCTCT (TC) n) located at 500 bp above the starting ATG codon of the *HMGA2* gene and correlates with the TC repeat length [30].

3. The fumaratehydratase gene (*FH*) encodes an enzyme in the tricarboxylic acid cycle and is associated with the development of hereditary leiomyomatosis. *FH* is a classic tumor suppressor gene, since all tumors caused by mutations in this gene exhibit somatic loss of the wild-type allele. Mutations in the *FH* gene stimulate the development of tumor LM in case of its biallelic inactivation; the mutative rate of this gene in patients that suffered from LM is 10.5% with biallelic loss of *FH* [31,32].

4. *COL4A5-COL4A6* genes are mapped on the long arm of chromosome X. The presence of specific deletions of the collagen genes is associated with diffuse leiomyomatosis. As determined by the screening, the 94 leiomyomas included 4 (4%) with a *COL4A5-COL4A6* deletion [32]. It is assumed that the driver of deletions in the *COL4A5* and *COL4A6* genes is the *IRS4* gene [32].

Mutations of the *MED12* gene and overexpression of the *HMGA2* gene have long been considered to be mutually exclusive, i.e., in different LMs changes (mutations or overexpression) of only one of these genes were detected [33]. The presence of two subtypes of LM was assumed: with mutations of the *MED12* gene (1) and with chromosomal rearrangements that affect the expression of the *HMGA2* gene (2). The real interaction of these genes in LM is more complicated [34]. A simultaneous analysis of mutations of the *MED12* gene and expression of the *HMGA2* gene yielded results indicating the possible interaction of the products of the corresponding genes in the pathogenesis of LM. In samples of myomatous nodes with a *MED12* gene mutation, almost half of the cases show increased expression of the *HMGA2* gene, which is also registered in myomas without *MED12* mutations [34]. The authors concluded that the increased expression of the *HMGA2* gene is most likely the primary link that launches the genetic program for the development of LM. It can be a consequence of various reasons, such as chromosomal rearrangement affecting the 12q15 region or hypomethylation of the *HMGA2* gene, which may be reflected in the combination of this process with the presence of mutations in the *MED12* gene. The secondary inducers of LM, most likely, can be other chromosomal rearrangements that do not affect chromosome 12, but directly or indirectly affect the expression of the *HMGA2* gene.

An important role in the pathogenesis of LM is played by the activation of Wnt/b-catenin and Wnt/MAPK metabolic signaling pathways [9]. It is these metabolic pathways that control the transformation of myometrial SC into connective tissue cells of fibroid LM, which, due to chaotic division and active synthesis of ECM, form definitive LM tumors. Proteins of the Wnt metabolic pathway, interacting with receptors of the Frizzled family (serpentine superfamily), activate the T-β-catenin protein and transcription factor (TCF), which produces transforming growth factor β (TGF-β)—the main inducer of excessive synthesis of extracellular matrix components [1].

In normal SCs, the *MED12* gene acts as a physiological modifier of β-catenin; however, when a mutation occurs, this function of the *MED12* gene is lost. Moreover, TGF-β receptors are expressed in SCs, which activate mitogenic protein kinase (MAPK) proteins, which regulate cell–cell interactions, renewal, and proliferation of SCs [10]. It is important to note that on the X chromosome near the *MED12* gene (up to 250 kb), a DNA sequence (rs5937008) was found that induces tumorigenesis and promotes the occurrence of mutations in the *MED12* gene. In tumors with the “high” risk allele (rs5937008), mutations in the *MED12* gene occurred significantly more often than in the absence of a risk allele [35]. The results of full-genome sequencing, taking into account the previously obtained data, suggest several different molecular mechanisms for the genesis of LM. Analysis of the expression of the genomic profile indicates disorders in the cells of LM in the main metabolic pathways: Wnt/β-catenin, prolactin, and insulin-like growth factor (IGF). In the case of changes in the *HMGA2* gene, LMs develop mainly due to activation of the *PLAG1* proto-oncogene and induction of the *WIF1* gene, an inhibitor of the Wnt/β-catenin metabolic pathway. The presence of mutations in the *MED12* gene and, in part, in the *RAD51B* gene (responsible for DNA repair and involved in the *HMGA2* gene) induces overexpression of the *SFRP1* gene, an inhibitor of the Wnt metabolic pathway. Biallelic inactivation of the fumaratehydratase gene causes activation of the oncogenic transcription factor NRF2 (*NFE2L2* gene). The *IRS4* gene is a LM driver for deletions in the *COL4A5* and *COL4A6* genes. *IRS4* encodes an insulin receptor substrate-4 that can stimulate proliferation by enhancing *IGF-1* function. These features of the molecular pathogenomics of LM should be considered in the treatment of LM [32]. In particular, in LM with changes in the *HMGA2* gene, activation of the insulin-like growth factor *IGF2BP2* gene occurs. Suppression of this signaling pathway is considered promising for the treatment of LM with overexpression of the *HMGA2* gene [36].

Molecular genetic studies of fibroids have provided new data on the clonal origin of this tumor and the nonrandom association of mutations with the type of tumor. In particular, it was found that in multiple uterine fibroids, *MED12* gene mutations occur twice as often as in solitary tumors (61% and 32%) [27]. Among patients with multiple LM, the *COMT* Val/Val genotype frequency is twice as high as among patients with solitary LM (40% and 20.3%) and among the controls (40% and 18.6%) [37]. Our molecular genetic studies have not yet allowed us to identify specific gene markers of LM, but expanded our understanding of the clonal development of the tumor. The data on the study of the X chromosome inactivation patterns on which the *MED12* gene is mapped, the exon 2 mutations of this gene, as well as the results of cytogenetic analysis convincingly proved that the LMs are of monoclonal origin; however, the clones of tumor cells in different myomatous nodes can be different, i.e., have different mutations that determine the individual characteristics of their molecular pathogenesis [25,38].

## 4. Genes Associated with the Development of LM

In addition to the group of genes considered, mutations of which can be a direct cause of the development of LM, hundreds of genes associated with various forms, severity, and characteristics of the clinical course of the disease have been identified. Most of them were detected by the GWAS method and by comparing the allelic frequencies of each candidate gene in normal individuals and in patients. The gene network of LM is represented by genes responsible for the metabolism of steroid hormones and their receptors, proliferation genes, cell contacts, angiogenesis, and growth factors, including oncogenes, pro-inflammatory cytokines and their suppressors, microRNA genes, and methylation [1,39]. Already, the first studies on functional mapping made it possible to confirm the association of LMs with more than 100 genes whose products are included in various metabolic pathways. Important genes for predisposition to LM are the genes for steroid hormones, cytokines, the immune response, and tumor growth factors [5]. At the same time, polymorphism of the *PROGINS* (progesterone receptor gene) according to individual authors is not a risk factor for the development of LM [40].

Important information on the pathogenomics of LM was obtained by analyzing the expression profiles of these genes. It was found that the level of estrogen, its ER-α receptor, and corresponding mRNA in LM cells were significantly higher than in normal myometrium [41]. Estrogens also contribute to the growth of LM, stimulating the synthesis of cytokines and growth factors, while blocking apoptosis by suppressing the *p53* gene [42].

Depending on the stage of development, localization, and type of tumor, gene expression of major growth factors (*EGF, HB-EGF, VEGF, bFGF, PDGF, TGF-b,* and *ADM*) varies significantly [43]. Epidermal growth factor (EGF), heparin-binding epidermal growth factor (HB-EGF), acidic fibroblast growth factor (aFGF), transforming growth factors-α and -β (TGF-α and TGF-β), main fibroblast growth factor (bFGF), and their receptors also play an important role in the development of LM. Isoforms of the proteins bFGF, VEGF, and TGF-b, even at low concentrations, significantly potentiate the proliferation of LM cells. An important role in the regulation of ECM volume is carried out by the transforming growth factor β (*TGF-β3)* gene, which significantly increases the synthesis of ECM proteins (fibronectin, collagens, and versicin) by suppressing the expression of genes that regulate the degradation of these proteins [44,45]. Other growth factors, such as FGF, are inducers of mitogenesis and differentiation of myometrial cells, including fibroblasts, SC, and vascular endothelial cells [43]. The cells of normal myometrium and LM significantly differ in the expression of angiogenesis genes. Thus, increased expression compared with the control is observed in LM cells for the *TGFB1* gene, and stably reduced for genes of connective tissue growth factor (CTGF) and cysteine-rich angiogenic inducer 61 (*CYR61*) [43].

Many cytokines, including tumor necrosis factor (TNF-α), interleukins (IL-1 and IL-6), as well as chemokines and their receptors (MIP-1 α, MIP-1β, RANTES, eotaxin, eotaxin-2, IL-8, CCR1, CCR3, CCR5, CXCR1, and CXCR2) are involved in the development of LM [11].

An important factor in the growth of LM, as already mentioned, is the extracellular matrix. In addition to the factors already mentioned that determine the growth of ECM, vitamin D plays a significant role in its development [46]. The main components of ECM (collagens, fibronectin, and proteoglycans) by LM cells are produced much more actively than normal myometrium cells [47]. A particularly important role in the formation of ECM is played by the growth factor with profibrotic activity—the transforming growth factor β. The *TGFB3* gene of the TGF-β3 subunit (signal mediator TGF-β) is overexpressed in LM cells [44].

Hundreds of candidate genes and dozens of metabolic pathways make up the LM gene network [5]. The latter includes embryonic developmental genes (*WNT* and *HOX*), *MED12*, steroid hormone metabolism genes and their receptors, collagen genes and ECM metabolism genes, many oncogenes and their suppressors, proliferation and growth factor genes, as well as apoptosis genes and their regulators. A comprehensive GWAS analysis of 15,453 patients with LM and 392,628 women in the control group revealed 22 loci in which 1428 SNPs were identified, and more than 30 candidate genes were found. The latter belong to two groups: 1) genes responsible for genomic stability (*TERT, TERC,* and *OBFC1*), including genes of telomeric regions of chromosomes—*TP53* and *AT*; and 2) genes that regulate the development of the urogenital system (*WNT4, WT1, SALL1, MED12, ESR1, GREB1, FOXO1,* and *DMRT1*), as well as the marker protein gene CD44 associated with mutations of the *MED12* gene.

The analysis of mutations and allelic variants of LM genes involved in the development of the female reproductive system is important for the search for new drugs and promising biomarkers of the disease. Thus, acid phosphatase genes *ACP1*, *PTH*, and *NRT2* are negatively associated with LM sizes. A small LM is characterized by a protective combination of alleles of the acid phosphatase *ACP1* gene and the non-receptor protein tyrosine phosphatase type 2 gene (Protein Tyrosine Phosphatase Non-Receptor Type ACP1 *B/* B-PTPN22 *C/*C) [48]. In LM cells, a decrease in the size of the telomeric regions of chromosomes and, correspondingly, a decrease in the expression of genes determining their sizes: *TERT*, *TERC*, and *OBFC1*, were noted. At the same time, telomere sizes in LM cells are smaller than in normal myometrium cells. A particularly significant decrease in telomeres is observed with unfavorable combinations of the alleles of the *TERT* (rs72709458, rs2736100, rs2853676), *TERC* (rs10936600), and *OBFC1* (rs1265164) genes [35]. Mutations and allelic variants of these genes, as well as chromosomal aberrations affecting their expression, create an “epigenetic landscape” that is unique for each patient and even for each tumor, in accordance with the type of LM that is formed and developed.

## 5. Epigenetic Regulation

In addition to heredity, an undoubted role in the genesis of LM is determined by epigenetic factors. All the main mechanisms of regulation of gene activity (DNA methylation, histone acetylation and methylation, regulatory micro-RNA, heterochromatization, and telomere shortening) in the pathogenomics of LM are often disturbed [9].

Numerous data have been obtained that indicate global disorders in methylation/demethylation of the genome in LM cells [49,50,51]. This indicates an important contribution of epigenetic regulation disorders to the pathogenesis of this disease [43,52]. An important role in the pathogenesis of LM is played by methylation of key embryonic development genes, namely *FOXO1*, *TERT*, and *WNT4* [35]. A significant role in the epigenetic control of the development of LM is played by regulatory miRNAs. Already, the first studies of the epigenetic mechanisms of LM showed significant disorders in the synthesis profile of regulatory microRNAs of the families let7, miR-21, miR-93, miR-106b, and miR-200 [53]. Many of them (let-7, 200a, 200c, 93, 106b, and 21) regulate proliferation, inflammation, angiogenesis, control the synthesis of ECM components, and apoptosis of LM cells [54]. Epigenetic changes in the LM genome activate important transduction (signal transmission) signaling pathways, such as Wnt/β-catenin and Wnt/MAPK. Currently, there are 12 known major metabolic pathways that are disrupted in LM cells [55]. They regulate the metabolism of steroid hormones, growth factors, transforming growth factor-beta, (TGFβ/Smad, Wnt/β-catenin), retinoic acid, vitamin D, and peroxisome receptors (PPAR). Some of these signaling pathways act cumulatively. For example, the metabolic pathways of MAPK and AKT, acting together, have a pronounced effect on the exchange of growth factors, hormones (estrogens), and vitamin D. Their analysis, clarification of the role of individual genes that determine their cumulative adverse effect, allows us to find new potential targets for the treatment of LM. As already mentioned, ECM and its components, which make up the bulk of the total mass of LMs, can become such a promising target.

Genes that regulate the development of the female reproductive system and genome stability determine a hereditary predisposition to LM [35]. It is believed that chromosomal instability, chromothripsis [51], as well as chromosomal translocations are the result of mutations in genome stability genes, DNA repair, and telomere size reduction. These same factors predispose an individual to the development of uterine tumors.

## 6. General Considerations

The novelty of this study is validating the position of LM on the decisive role of ECM in increasing tumor mass and the associated factors, as well as, according to some experts, the need to shift the center for targeted therapy of this disease from the myomatous cell component of LM to suppress the growth of the ECM mass [16].

There is no doubt that among the factors involved in the tumor degeneration of stem/progenitor cells, the *MED12* and *HMGA2* genes play an important role, the pathological effect of mutations of which is realized through dysfunction of various metabolic pathways, primarily Wnt/ß-catenin and Wnt/MAPK, and also prolactin and insulin-like growth factor (IGF) [32]. Important information on the molecular mechanisms of the pathogenesis of LM was obtained by analyzing the transcriptional profile of LM cells with various driver mutations. Already the first studies have established that when the expression of the *HMGA2* gene is changed, LMs develop mainly due to the activation of the *PLAG1* proto-oncogene and the induction of the *WIF1* gene, an inhibitor of the Wntβ-catenin metabolic pathway. Mutations in the *MED12* gene and, in part, in the *RAD51B* gene (responsible for DNA repair and involved in the *HMGA2* gene) cause overexpression of the *SFRP1* gene, an inhibitor of the Wnt metabolic pathway, and activation of the oncogenic transcription factor NRF2 (*AKR1B10* gene) in the fumarase gene.

The results of the transcriptome analysis confirm and complement the data from the study of fibroids with various gene mutations. For a long time, it was believed that either mutations of the *MED12* gene or overexpression of the *HMGA2* gene can occur in LM. It has recently been found that LM cells with a *MED12* gene mutation may also have increased expression of the *HMGA2* gene, i.e., the products of these genes are possibly functionally conjugated [34]. Indirect data have been obtained showing that a certain number of dinucleotide repeats (TC-27) in the promoter part of the *HMGA2* gene are associated with overexpression of this gene in LM cells [30], which, possibly, leads to the appearance and growth of the LM node. A change in the number of dinucleotide repeats in the *HMGA2* gene can induce an abnormal cell response to damaging factors: Mechanical transduction associated with periodic myometrial remodeling. The action of the growing intercellular matrix may contribute to the appearance of chromosome rearrangements, including chromosome 12, and, as a result, lead to overexpression of the *HMGA2* gene. In LM cells with altered *HMGA2* gene expression, the insulin-like growth factor *IGF2BP2* gene is activated, and therefore suppression of this signaling pathway is considered promising for the treatment of LM with *HMGA2* mutation [36].

Chromosomal rearrangements are typical for single large nodes of LM, while for multiple LMs, the presence of *MED12* gene mutations is more characteristic. Their presence in almost 70% of patients with LM, of course, cannot be attributed only to spontaneous somatic mutagenesis. It is believed that one of the reasons for the high mutability of exon 2 of the *MED12* gene is the presence of a specific high-risk site (rs5937008) located 250 kb above the *MED12* gene [35]. The mechanism of its action is unknown and, possibly, is associated with the selection of LM cells without the *MED12* mutation, which carry the “high risk” allele. It is possible that cells with a mutation in the *MED12* gene, due to overexpression of the *SFRP1* gene, an inhibitor of the Wnt metabolic pathway or other metabolic changes associated with impaired mutant gene, gain additional survival benefits. The molecular mechanism for selecting cells with a *MED12* gene mutation remains unknown.

The results of studying the distribution characteristics of *MED12* mutations, X-chromosome inactivation patterns on which the *MED12* gene is mapped, as well as chromosome analysis data convincingly prove that LM is a monoclonal disease; however, tumor cell clones in different myomatous nodes may be different, i.e., have different mutations that determine the individual characteristics of molecular pathogenesis.

A clear correlation was found between the frequency and types of mutations, as well as the frequencies of alleles and genotypes of a number of genes, one of which is the *COMT* gene, with the development of multiple and single myomas [37], as well as a combination of alleles of the *ACP1* gene and *PTPN22* gene with LM sizes [48].

It has been established that microRNAs are the regulators of LM growth, and, first of all, miR-15b, which increases the expression of genes that control cell proliferation and affects the receptivity of estrogen and progesterone [50].

Important information on the pathogenomics of LM has been obtained in recent years using a genome-wide analysis of allelic associations and analysis of candidate gene expression. Two large-scale population studies of LM revealed the presence of 22 loci associated with LM, and identified dozens of genes responsible for maintaining the integrity of the genome, including genes that determine telomerase synthesis and sizes of telomeric regions of chromosomes, a large group of genes associated with malignant and benign hormone-dependent tumors and genes that control the embryonic development of the female reproductive system [35,56].

**Figure 1:**
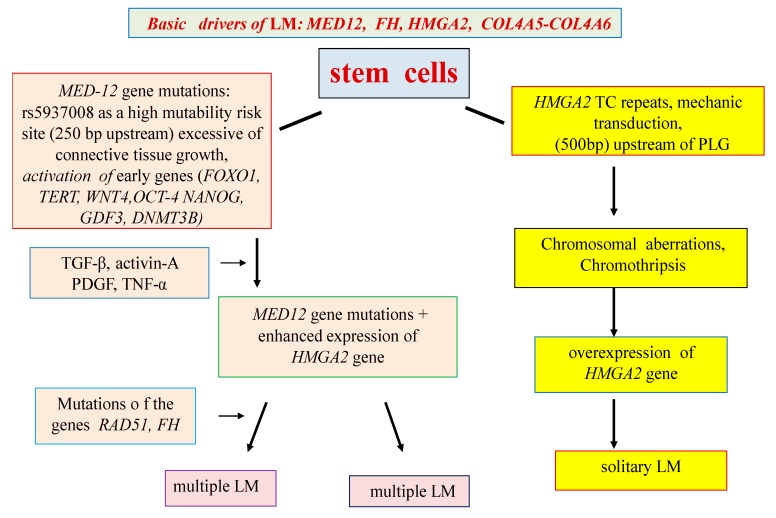
Hypothetical scheme for solitary and multiple LM development.

The pathogenetic mechanisms of interaction of numerous candidate genes and metabolic pathways of LM have not yet been studied sufficiently and require clarification. At the same time, it seems likely that the presence of various types of LM and the characteristics of the clinical and pathomorphological manifestations of the disease are determined not only by the main driver mutations, but also by numerous candidate genes that are expressed starting from the earliest stages of the tumor, possibly, in some cases, in utero (Figure 1).

## 7. Conclusions

Recent studies have significantly expanded our understanding of the pathogenomics of LM, particularly regarding the parental (progenitor) cells of LM, the gene networks of the disease, the features of intergenic interactions, and the functions of the main driver mutations that potentiate the development of LM. New strategic therapies have been outlined.

A review of the available data suggests the presence of at least two mechanisms for the development of LM: genetic and epigenetic. Most likely, the primary link in the LM genetic program is the mutations of the *MED12* gene, which arise in the SC of the uterine myometrium under the influence of unfavorable factors (infections, mechanotransduction, etc.). The mutations result in metabolic pathway dysfunctions associated with the proliferation of myoblasts and the formation of an extracellular matrix (Wnt/β-catenin, prolactin, and insulin-like growth factor—IGF). The results of these abnormalities are multiple medium-sized LM nodes According to the epigenetic hypothesis, the main driving force (driver) of LM is overexpression of the *HMGA2* gene, caused by many external factors (muscle contraction, hypoxia, disorders of the xenobiotic detoxification system, chromosomal aberrations, etc.). Overexpression of the *HMGA2* gene leads to activation of the *PLAG1* proto-oncogene and induction of the *WIF1* gene, an inhibitor of the Wnt/β-catenin metabolic pathway. The results of epigenetic disorders are usually large solitary LM.

The pathogenomics of LM opens up new strategic paths for the prevention, diagnosis and treatment of this disease. In particular, suppression of the signaling pathway associated with the activation of the insulin-like growth factor *IGF2BP2* gene is promising for the treatment of fibroids with overexpression of the *HMGA2* gene. Given the crucial role of ECM in the development of LM, effective control of its size (suppression of the *TGF-β* and *ACVR1* genes) is considered as a new promising strategy for the pharmacotherapy of LM.

The identification of candidate genes for LM and unfavorable combinations of minor alleles and their epigenetic regulation opens up new possibilities for assessing the risk of developing LM and the features of its clinical manifestation. In so doing, one can also develop approaches for early prevention and new methods of treatment for this socially significant gynecological disease.

## Abbreviations:

GWAS	genome-wide association studies
LM	leiomyoma
BrdU	5-bromo-2–deoxyuridine
SC	stem cells
ECM	extracellular matrix
ERα	estrogen receptor α
PR	progesterone
FH	fumaratehydratase gene
TCF	transcription factor
TGF-β	transforming growth factor β
MAPK	mitogen-activated protein kinase
IGF	insulin-like growth factor
EGF	epidermal growth factor
HB-EGF	heparin-binding epidermal growth factor
CGH	comparative genomic hybridization
